# Effects of Whole-Body Vibration on Exercise Performance among Athletes: A Systematic Review and Meta-Analysis of Randomized Controlled Trials

**DOI:** 10.5114/jhk/193514

**Published:** 2024-12-19

**Authors:** Yu-Ching Peng, Yi-Ting Guo, Jeng-Cheng Wu, Wen-Hsuan Hou

**Affiliations:** 1 School of Medicine, College of Medicine, Taipei Medical University, Taipei, Taiwan.; 2 Department of Medical Education, Taipei Medical University Hospital, Taipei, Taiwan.; 3 Department of Medical Education, Taipei Veterans General Hospital, Taipei, Taiwan.; 4 Department of Urology, Taipei Medical University Hospital, Taipei, Taiwan.; 5 Taipei Medical University–Research Center of Urology and Kidney, Taipei Medical University, Taipei, Taiwan.; 6 Department of Education and Humanities in Medicine, School of Medicine, College of Medicine, Taipei Medical University, Taipei, Taiwan.; 7 Department of Health Promotion and Health Education, College of Education, National Taiwan Normal University, Taipei, Taiwan.; 8 Department of Physical Medicine and Rehabilitation, School of Medicine, College of Medicine, Taipei Medical University, Taipei, Taiwan.; 9 Cochrane Taiwan, Taipei Medical University, Taipei, Taiwan.; 10 Graduate Institute of Clinical Medicine, College of Medicine, Taipei Medical University, Taipei, Taiwan.; 11 School of Gerontology and Long-Term Care, College of Nursing, Taipei Medical University, Taipei, Taiwan.; 12 Department of Physical Medicine and Rehabilitation, Taipei Medical University Hospital, Taipei, Taiwan.

**Keywords:** power, strength, aerobic endurance, sport performance, youth athletes

## Abstract

Whole-body vibration (WBV), an intervention for enhancing athletes’ exercise performance (muscle strength and power), is often used either as a supplement or an alternative to conventional training. The current systematic review and meta-analysis assessed the effects of WBV on exercise performance in athletes. PubMed, Embase, and Cochrane Library databases were searched for relevant randomized controlled trials published from database inception to April 2024. We analyzed three key components of exercise performance: muscle power (measured in terms of countermovement jump (CMJ) and squat jump (SJ) height), strength (measured in terms of isometric and concentric torque of the knee extensors and flexors), and aerobic cardiovascular endurance (measured in terms of maximal oxygen uptake (VO_2max_)). This review included 18 randomized controlled trials. WBV significantly improved concentric torque of the knee extensors and flexors, with effect sizes of 8.86 (95% confidence interval: 6.00 to 11.72; I^2^ = 0%; p < 0.00001) and 9.56 (95% confidence interval: 7.40 to 11.72; I^2^ = 0%; p < 0.00001), respectively. However, no significant changes were noted in the indicators of muscle power or cardiovascular endurance. Overall, our findings suggest that WBV interventions can enhance lower-limb strength in athletes. However, the quality of the evidence was low. To provide effective evidence-based guidance for WBV, future studies should consider participants’ characteristics as well as intervention frequency, intensity, and duration in their analysis.

## Introduction

Vibration exercises typically involve whole-body vibration (WBV), a passive exercise modality in which mechanical stimuli from a vibrating platform are transmitted through the body (Mansfield, 2005). WBV is believed to improve muscle strength. Research indicates that vibratory stimulation can enhance neuromuscular activity, which was confirmed through surface electromyography (Ritzmann et al., 2010). A systematic review concluded that WBV protocols with higher frequencies and amplitudes were more effective in increasing muscle strength (Al Masud et al., 2022). However, the literature has reported inconsistent findings regarding the benefits of WBV, likely because of variations in the intervention type, frequency, amplitude, and duration across studies (Cochrane et al., 2009).

Evidence suggests that WBV offers a range of benefits. It enhances strength and flexibility (Jacobs and Burns, 2009) as well as trunk muscle strength (Maeda et al., 2016) in healthy adults. WBV was demonstrated to improve neuromuscular performance and muscle strength, and thus balance and postural control, in athletes with ankle instability (Coelho-Oliveira et al., 2023). This intervention also supports muscle function through the tonic vibration reflex (TVR), improves muscle energy metabolism through vibration-induced muscle contractions, and increases the muscle perfusion rate and temperature to enhance power (Chena Sinovas et al., 2015; Till et al., 2017).

Similarly to aerobic exercise, WBV may improve cardiovascular endurance (Park et al., 2015). However, whether WBV causes sufficient stimulation to improve cardiorespiratory fitness remains to be determined. Activities such as strength training improve cardiorespiratory fitness by enhancing vascular function, increasing the muscle blood flow, and promoting skeletal muscle adaptations. These adaptations, including vibration-induced muscle hypertrophy, may improve oxygen transport and utilization, thereby enhancing VO_2max_ (Bogaerts et al., 2009). A study indicated that incorporating WBV into exercise regimens confers incremental advantages by enhancing functional capacity, expanding thoracic mobility, and facilitating rehabilitation in patients with a stroke and respiratory dysfunction (Duray et al., 2023). A meta-analysis reported that WBV benefits patients with chronic obstructive pulmonary disease by improving their functional exercise capacity without causing any adverse effects (Cardim et al., 2016).

Limited data are available regarding the effects of WBV on exercise performance in athletes. Some studies have reported that WBV improves lower-limb muscle strength. For example, Annino et al. (2007) demonstrated that short-term WBV improved explosive knee extensor strength in elite ballet dancers. Arora et al. (2021) indicated that WBV, as an additional modality, could enhance the neuromuscular activity of lower-limb muscles in athletes. Colson et al. (2010) concluded that incorporating short-term WBV into preseason training for basketball players increased their knee extensor strength and squat jump (SJ) performance. Karatrantou et al. (2013) reported that WBV enhanced knee flexor mobility and strength in moderately active women. Egesoy and Yapıcı (2023) demonstrated that a 6-week WBV program improved vertical jump and sprint performance in volleyball players.

Unlike the aforementioned studies, a study by Kvorning et al. (2006) revealed that compared with conventional resistance training alone, a combination of WBV with conventional resistance training led to no additional improvements in maximal isometric voluntary contraction or mechanical performance metrics (e.g., jump height, mean power, peak power, and peak velocity) in the countermovement jump (CMJ). Similarly, Bertuzzi et al. (2013) reported that adding WBV to strength training did not significantly improve dynamic strength in long-distance runners. Furthermore, in the study of Martinez-Pardo et al. (2013), WBV at different amplitudes resulted in no significant alterations in vertical jump variables such as height, peak power, and the force development rate.

Several systematic reviews and meta-analyses have explored the effects of WBV. A systematic review reported that WBV could improve muscle strength, power, and flexibility (Alam et al., 2018). However, this review did not focus on athletes. Moreover, no meta-analysis was performed because the review included only five studies. A meta-analysis concluded that WBV exerted only small and inconsistent effects on the short- and long-term performance of competitive and elite athletes (Hortobagyi et al., 2015). However, that analysis was conducted in 2015, and several randomized controlled trials (RCT) have been published since then. Another meta-analysis reported that WBV exerted no significant short- or long-term effects on jump performance, sprint performance, or agility in athletes or physically active individuals (Minhaj et al., 2022). However, that study imposed language restrictions during the literature search and did not explore aspects of athletic performance such as muscle strength and cardiovascular endurance.

Considering the aforementioned background, the current systematic review and meta-analysis of RCTs evaluated the effects of WBV on three major components of exercise performance in athletes: power, strength, and cardiovascular endurance.

## Methods

### Information Sources

The protocol for this study was registered in PROSPERO (CRD42022371545). The findings are reported in accordance with the Preferred Reporting Items for Systematic Reviews and Meta-Analyses (PRISMA) guidelines (Appendix 1A and Appendix 1B) (Page et al., 2021). A librarian of our research team systematically searched multiple databases for relevant articles published from database inception to April 2024.

### Eligibility Criteria

The inclusion criteria were as follows: having an RCT design, involving healthy adult (15–40 years, excluding children) athletes of any sex or race who professionally or recreationally engaged in sports (e.g., ballet, basketball, sprinting, and volleyball), and assessing the effects of WBV. The search was not restricted by language. We included RCTs in which WBV was implemented (as part of exercise training) for at least two weeks (Ashton et al., 2020). Although a recent study showed acute WBV could activate either the sympathetic or the parasympathetic nervous system (Tan et al., 2024), evidence suggests that a minimum of two weeks of training can improve heart rate reserve and maximum oxygen uptake (Borresen and Lambert, 2008).

The exclusion criteria were as follows: including individuals with comorbidities or those with previous experience in WBV, having a complex WBV protocol, involving a combination of vibration types, assessing only the immediate effects of only one or few sessions of WBV, and having a single-arm or a crossover design.

The inclusion criteria were defined using the following Participant, Intervention, Comparator, Outcome, Time, and Study design framework. Participants were healthy athletes who professionally or recreationally engaged in any sports. The intervention was WBV, including synchronous vertical (SV) or side-alternating (SA) vibrations. The control group received conventional training without WBV. The outcomes were CMJ height, SJ height, isometric and concentric torque of the knee extensors and flexors, and VO_2max_ during aerobic exercise. The intervention duration was >2 weeks. The target study design was the RCT.

### Search Strategy

Two reviewers (Y.-C.P. and Y.-T.G.) used the keywords “whole body vibration” AND (“athletes” OR “sports” OR “exercise”) to search for relevant RCTs in the independently searched PubMed, Embase, Cochrane Library, and Chinese Electronic Periodical Services (CEPS) databases using appropriate keywords for relevant articles published from database inception to April 20, 2024. In addition, ISRCTRN Registry, Clinicaltrials.gov, and Open Science Framework were searched for articles published during the same period. The search strategy is presented in Appendix 2.

### Selection and Data Collection Processes

The same two researchers independently evaluated all retrieved abstracts, studies, and citations and subsequently discussed their findings. Any disagreements were resolved through discussion with a third reviewer (W.-H.H.). Y.-C.P. and Y.-T.G. independently extracted the following data: the study country, the sport type, sample size, athlete age, interventions in the experimental and control groups, and WBV protocols and variables (e.g., vibration type, peak-to-peak displacement, and the body position). Of the potentially eligible articles, we identified and removed duplicate publications and those that did not have full-text availability. At the title-abstract stage of screening for eligibility (stage I), we excluded articles on the basis of their titles and abstracts, respectively. At the full-text stage of screening for eligibility (stage II), we excluded several articles because of the following reasons: did not enroll athletes, did not use a parallel design, had intervention duration of <14 days, or assessed outcome indicators not measured by any other study.

### Data Item

To evaluate the effects of WBV training, we measured the athletes’ exercise performance before and after the intervention. Seven indicators were used to assess three key components of exercise performance: CMJ and SJ height (power), isometric and concentric torque of the knee extensors and flexors (strength), and VO_2max_ during aerobic exercise (cardiovascular endurance).

### Study Risk of Bias Assessment

Two reviewers (Y.-C.P. and Y.-T.G.) independently assessed the risk of bias using the revised Cochrane risk-of-bias tool for RCTs (version 2; Bristol, England, UK) (Sterne et al., 2019). This tool can be used to evaluate risks of bias in six distinct domains: the randomization process, deviations from intended interventions, missing outcome data, outcome measurement, result selection, and overall bias. Each domain includes specific questions for measuring potential bias, with response options of “yes”, “probably yes”, “probably no”, “no”, and “no information”. In this study, each domain was categorized as having a low risk of bias, a high risk of bias, or some concerns. Any between-reviewer disagreements were resolved through discussions with a third author (W.-H.H.). The Grading of Recommendations, Assessment, Development, and Evaluation (GRADE) criteria were used to evaluate the certainty of evidence for the study outcomes (Guyatt et al., 2011).

### Effect Measures and Synthesis Methods

All statistical analyses were performed using a random-effects model in RevMan (version 5.4.1), which offers the statistical program MetaView for data visualization. Mean difference and 95% confidence interval (CI) values were calculated for each RCT and are presented in forest plots. If the standard deviation for post-intervention changes or the actual correlation coefficient was not reported, a correlation coefficient of 0.8 was used to estimate the standard deviation for changes from baseline (Higgins et al., 2019). To assess heterogeneity, *I*^2^ statistics were calculated; an *I*^2^ value of >50% indicated substantial heterogeneity. The potential small-study bias was visually examined using funnel plots (Song et al., 2002). Significance was set at *p* < 0.05; for publication bias, the threshold was set at *p* < 0.10.

For articles presenting only graphical data, values were estimated from figures using WebPlotDigitizer (version 4.5) (Rohatgi, 2023). Data were analyzed using RevMan (version 5.4.1; Cochrane Collaboration, Oxford, UK).

The present study did not involve human participants and thus did not require Institutional Review Board approval.

## Results

### Study Selection

**Figure 1:**
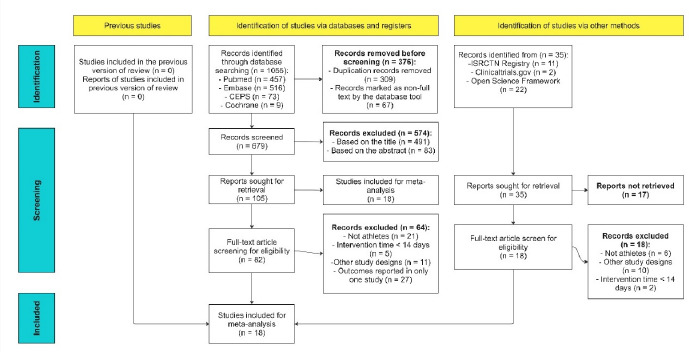
Flowchart depicting article selection. The review process adhered to Preferred Reporting Items for Systematic Reviews and Meta-Analyses guidelines.

Figure 1 presents a flowchart (following the Preferred Reporting Items for Systematic Reviews and Meta-Analyses 2020 guidelines (PRISMA 2020)) depicting the processes of article screening and selection. As mentioned, we also searched the grey literature, which included preprint articles, conference abstracts, study register entries, clinical study reports, dissertations, unpublished manuscripts, government reports, and any other documents providing relevant information. Of the potentially eligible articles, 376 were excluded before screening because they were duplicate publications (309) or they did not have full-text availability (67). At the title-abstract stage of screening for eligibility (stage I), we excluded 491 and 83 articles on the basis of their titles and abstracts, respectively. Moreover, 23 articles could not be retrieved and were thus excluded. At the full-text stage of screening for eligibility (stage II), we excluded 64 RCTs because of the following reasons: 21 did not enroll athletes, 11 did not use a parallel design, 5 had intervention duration of <14 days (insufficient period for noticeable changes in outcomes), and 27 assessed outcome indicators not measured by any other study. Finally, 18 studies meeting the inclusion criteria were considered in our meta-analysis.

### Study Characteristics

Table 1 presents the characteristics of the included studies. Table 2, Appendix 3 and Appendix 4 present the characteristics of the WBV protocols used in the included studies. The presentation adhered to the recommendations of the International Society of Musculoskeletal and Neuronal Interactions (Rauch et al., 2010). The 18 RCTs were published between 2005 and 2022 (Annino et al., 2007; Arora et al., 2021; Bertuzzi et al., 2013; Celik et al., 2022; Cheng et al., 2012; Delecluse et al., 2005; Di Giminiani et al., 2009; Fagnani et al., 2006; Fort et al., 2012; Karatrantou et al., 2013; Kvorning et al., 2006; Lamont et al., 2011; Martinez-Pardo et al., 2013; Oosthuyse et al., 2013; Roschel et al., 2015; Rubio-Arias et al., 2018; Wang et al., 2014). The sample sizes of the studies ranged from 14 to 38. Our analysis included 392 athletes (intervention group: 208; control group: 184). The intervention duration ranged from 3 to 15 weeks. Most studies used synchronous vertical vibrations (n = 14) or side-alternating vibrations (n = 2). Two RCTs did not provide detailed information on the vibration type. Our study focused primarily on young athletes. The settings used for our analysis were based on the included RCTs.

**Table 1:** Characteristics of the included studies.

Author(s)	Publication year	Country	Sport type	Total (M/F)	Age (Y ± SD)	EG/CON (n)	Intervention other than WBV in EG	Intervention in CON	Outcome
Annino et al.	2007	Italy	Ballet	22(NA/NA)	21.2 ± 1.46	11/11	ballet training	ballet training	CMJ
Arora et al.	2021	India	Football, basketball, and volleyball	23 (23/0)	21.9 ± 1.87	12/11	squats and heel raise	squats and heel raise	CMJ
Bertuzzi et al.	2013	Brazil	Running	16(NA/NA)	32.5 ± 5.52	8/8	half-squat training sessions	half-squat training sessions	VO_2max_
Celik et al.	2022	Turkey	Basketball	16 (16/0)	15.5± 0.41	8/8	standard basketball training drills and skills	standard basketball training drills and skills	SJ, CKE, and CKF
Cheng et al.	2012	Taiwan	Volleyball, tennis, taekwondo, and track and field	23 (23/0)	20.1 ± 1.61	11/12	isometric semisquat training protocol (static semisquat position )	isometric semisquat training protocol (static semisquat position )	IKE, IKF, and VO_2max_
Colson et al.	2010	France	Basketball	18 (13/5)	19.9 ± 1.5	10/8	regular training routines	regular training routines	CMJ, SJ, and IKE
Delecluse et al.	2005	Brazil	Sprinting	20 (13/7)	21.2 ± 3.85	10/10	regular training routines	regular training routines	CMJ, IKE, IKF, CKE, and CKF
Fagnani et al.	2006	Italy	Volleyball, basketball, track and field, and gymnastics	24 (0/24)	23.8 ± 1.86	13/11	regular training routines	regular training routines	CMJ
Fort et al.	2012	Spain	Basketball	23 (0/23)	15.8 ± 1.15	12/11	regular training routines	regular training routines	CMJ
Giminiani et al.	2009	Italy	Gymnastic, swimming, and track and field	21 (11/10)	21.9 ± 1.4	10/11	NA	NA	CMJ and SJ
Karatrantou et al.	2013	Greece	moderately active physical activities	26 (0/26)	20.4 ± 0.4	13/13	NA	usual activities	CMJ, SJ, IKE, IKF, CKE, and CKF
Kvorning et al.	2006	Denmark	moderately trained sport activities	19 (19/0)	23.4 ± 1.25	10/9	weight loaded squat	weight loaded squat	CMJ
Lamont et al.	2008	USA	Recreational resistance training	24 (24/0)	23.6 ± 0.9	13/11	squat traininig program	squat traininig program	SJ
Martínez-Pardo et al.	2013	Spain	Recreational activities	38 (30/8)	21.2 ± 4	27/11	regular training routines	regular training routines	CMJ and SJ
Oosthuyse et al.	2013	South Africa	Road cycling	17 (12/5)	40.5 ± 8.98	9/8	regular cycling training routines	regular cycling training routines	VO_2max_
Roschel et al.	2015	Brazil	Recreational running	15(NA/NA)	32.7 ± 6.68	7/8	resistance training	resistance training	VO_2max_
Rubio-Arias et al.	2018	Spain	Recreational activities	33 (33/0)	23.2 ± 5.64	17/16	static contractions	static contractions	CMJ
Wang et al.	2014	Taiwan	Track and field	14 (14/0)	20.8 ± 1.56	7/7	loading training	load training	IKE

M, male; F, female; Y, year; SD, standard deviation; EG, experimental group; CON, control group; WBV, whole-body vibration; NA, data unavailable; CMJ, countermovement jump; VO_2max_, maximal oxygen uptake; SJ, squat jump; CKE, concentric knee extension; CKF, concentric knee flexion; IKE, isometric knee extension; IKF, isometric knee flexion

**Table 2:** Protocols and variables related to the WBV intervention.

Author	Device and brand name	Vibration type	Vibration frequency (Hz)	Peak-to-peak displacement (mm)	Peak acceleration (*g*)	Support device	Type of footwear
Annino et al.	the Nemes LC device	SV	30	5	5	none	dancer-type shoes
Arora et al.	NA	NA	20–25 to 30–35 to 40	NA	NA	none	NA
Bertuzzi et al.	Power Plate, IL	SV	12–45	1.7–5	NA	NA	sport shoes
Celik et al.	Power Plate, IL	SV	35	2	4.93	NA	sport shoes
Cheng et al.	AV-001A; Body Green Technology Co. Ltd.	SV	30	1–2	NA	NA	non-slippery socks
Colson et al.	Silverplatine first generation, Silver® Développement	SV	40	4	NA	with arms akimbo	socks
Delecluse et al.	Power Plate, IL	SV	35–40	1.7–2.5	NA	NA	NA
Fagnani et al.	Nemes LCB-040	SV	35	4	17	hands on the hips	gymnastic shoes
Fort et al.	Nemes Bosco Platform, Byomedic	SV	25–35	4	NA	none, with arms akimbo or holding balls	sport shoes
Giminiani et al.	Nemes-Lsb, Bosco-System	SV	20–55	2	5.5	holding railing	NA
Karatrantou et al.	Galileo Fitness, Novotec	SA	25	6	NA	NA	non-slippery socks
Kvorning et al.	Galileo 2000, Novotec Maschinen GmbH	SA	20–25	4	NA	NA	barefoot
Lamont et al.	Power Plate Next Generation, IL	NA	50	4 to 6	NA	holding the handles	NA
Martínez-Pardo et al.	Power Plate Next Generation, IL	SV	50	2, 4	NA	NA	NA
Oosthuyse et al.	Power Plate Next Generation, IL	SV	30	4	5	holding railing	sports shoes
Roschel et al.	Power Plate, IL	SV	30–50	6	NA	NA	sport shoes
Rubio-Arias et al.	Fitvibe Medical	SV	30–45	2–4	NA	NA	NA
Wang et al.	synchronous vibration platform, custom designed by Magtonic, Corp.	SV	30	4	10.68–10.9	With arms akimbo or holding the barbell	barefoot

WBV, whole-body vibration; NA, data unavailable; SV, synchronous vertical; SA, side-alternating

### Risk of Bias in Included Studies

**Figure 2:**
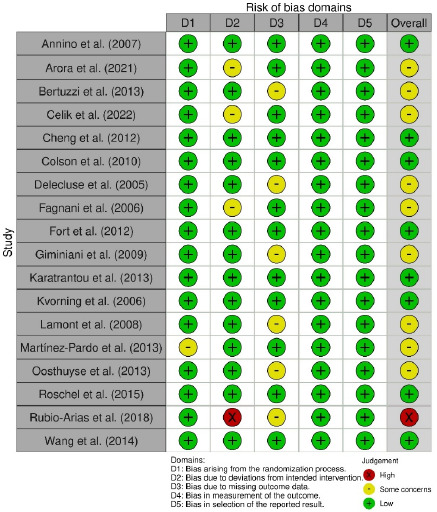
Results of risk-of-bias assessment.

Regarding the outcome exercise performance, our risk-of-bias analysis indicated a low risk of bias for eight RCTs, some concerns for nine RCTs, and a high risk of bias for one RCT (Figure 2). Specifically, in domains 1–3, some concerns were noted for one, three, and six RCTs, respectively, because of factors such as nonrandomized group assignment, a single-blind study design, intragroup differences in exercise, and participant attrition. All 18 RCTs had a low risk of bias in the outcome measurement and result selection domains. Because the assessors were blinded in all RCTs, the studies were considered to have a low risk of detection bias. Studies that reported unadjusted, raw data for outcomes were regarded as having a low risk of reporting bias.

### Results of Particular Studies

We measured seven indicators of exercise performance in athletes: CMJ height and SJ height for power, isometric and concentric torque of the knee extensors and flexors for strength, and VO_2max_ during aerobic exercise for cardiovascular endurance.

**Figure 3:**
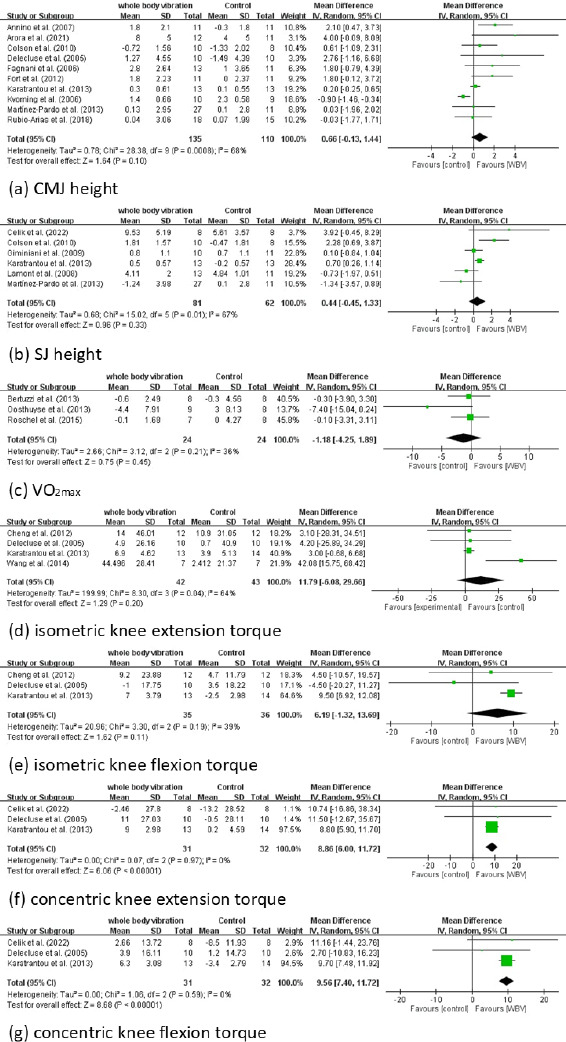
Forest plots for included studies.

The pooled effect size of 10 RCTs for CMJ height was 0.66 cm higher in the intervention than in the control group (95% Cl: −0.13 to 1.44; *p* = 0.1; *I^2^* = 68%). The pooled effect size of five RCTs for SJ height was 0.44 cm higher in the intervention than in the control (95% Cl: −0.45 to 1.33; *p* = 0.33; *I^2^* = 67%). However, the between-group differences were non-significant for both variables. Furthermore, moderate heterogeneity was observed for these variables (Figure 3a,b).

The pooled effect size of three RCTs for VO_2max_ was −1.18 (95% Cl: −4.25 to 1.89; *p* = 0.45; *I^2^* = 36%). Therefore, improvement in oxygen uptake was smaller in the intervention than in the control group; however, the between-group difference was non-significant (Figure 3c).

The pooled effect size of four RCTs for isometric torque of the knee extensor was 11.79 N•m (95% CI: −6.08 to 29.66; *p* = 0.20; *I*^2^ = 64%). The pooled effect size of three RCTs for isometric torque of the knee flexors was 6.19 N•m (95% CI: −1.32 to 13.69; *p* = 0.11; *I*^2^ = 39%). Therefore, the intervention and control groups did not differ significantly in terms of changes in the isometric torque of the knee extensors and flexors (Figure 3d,e).

The pooled effect sizes of three RCTs for concentric torque of the knee extensors and flexors were 8.86 N•m (95% CI: 6.00 to 11.72; *p* < 0.00001; *I*^2^ = 0%) and 9.56 N•m (95% CI: 7.4 to 11.72; *p* < 0.00001; *I*^2^ = 0%), respectively, higher in the intervention than in the control group. Notably, no heterogeneity was observed for these variables (Figure 3f,g).

### Results of Syntheses

**Figure 4:**
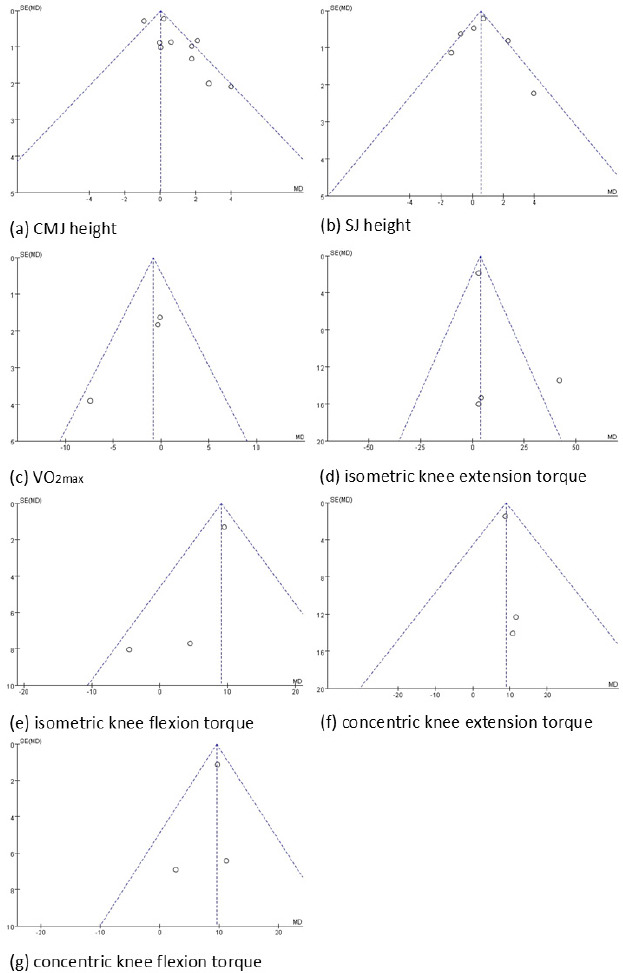
Funnel plots for included studies.

Funnel plots for the seven indicators used in this study are presented in Figure 4. No significant heterogeneity was observed for VO_2max_, concentric torque of the knee extensors or flexors, or isometric torque of the knee flexors. However, for the two indicators of power (CMJ height and SJ height) and one indicator of strength (isometric torque of the knee extensors), some heterogeneity was observed, with distribution beyond the two diagonal lines (Song et al., 2002).

The GRADE results are presented in Appendix 5. Overall, the quality of evidence supporting the positive effects of WBV on VO_2max_, isometric torque of the knee flexors, and concentric torque of the knee extensors and flexors was low. Moreover, the quality of evidence supporting the positive effects of WBV on CMJ height, SJ height, and isometric torque of the knee extensors was very low.

## Discussion

### Overall Effects

To the best of our knowledge, this is the first meta-analysis of the effects of WBV on three major components of exercise performance in athletes: power, strength, and cardiovascular endurance. WBV has been extensively studied for its potential to enhance athletic performance (Alam et al., 2018; Coelho-Oliveira et al., 2023; Hortobagyi et al., 2015; Minhaj et al., 2022).

Mechanical vibration applied during WBV induces cyclic transitions between eccentric and concentric muscle contractions, eliciting a neuromuscular response (Rauch et al., 2010). WBV has been reported to enhance muscle strength, power, and endurance (Alam et al., 2018). The WBV-mediated increase in muscle activity is mediated by several neural mechanisms, such as hormonal factors, TVR activation, and alterations in proprioceptor discharge (Da Silva-Grigoletto et al., 2009). Among these, the most commonly reported mechanism is TVR activation, which occurs during direct vibratory musculotendinous stimulation (Nordlund and Thorstensson, 2007). The TVR, triggered by vibratory stimulation of muscle spindles’ Ia fibers, leads to muscle contractions (De Gail et al., 1966; Hagbarth and Eklund, 1966) through both monosynaptic and polysynaptic pathways (Desmedt and Godaux, 1978; Matthews, 1966).

Physiologically, WBV is theorized to enhance exercise performance by stimulating muscle contractions (Rigoni et al., 2022; Ritzmann et al., 2010). This stimulation may enhance muscle activation, thereby increasing muscle strength (Masud et al., 2022). Some researchers have suggested that WBV expedites recovery by reducing muscle soreness and improving metabolic waste clearance from muscles after intense exercise (Herrero et al., 2011; Mahbub et al., 2019). Regarding cardiovascular endurance, researchers have proposed that WBV increases metabolic activity by increasing muscular activity (Rittweger et al., 2001). This effect is similar to the increase in the heart rate and lactate concentration that occurs during aerobic exercise (Crevenna et al., 2003).

Empirical studies have demonstrated the positive effects of WBV on athletes’ lower-limb muscle strength (Colson et al., 2010; Wang et al., 2014). For example, Annino et al. (2007) reported that short-term WBV effectively improved explosive knee extensor strength in elite ballet dancers. Conversely, Kvorning et al. (2006) reported that compared with conventional resistance training alone, a combination of WBV and conventional resistance training led to no additional improvements in maximal voluntary isometric contraction and mechanical performance. A meta-analysis suggested that WBV effectively increased lower-limb strength, but not upper-limb strength, lower-limb power or overall muscle endurance (Gonçalves de Oliveira et al., 2023). Another meta-analysis highlighted the lack of sufficient evidence supporting the positive effects of WBV on neuromuscular performance of individuals with spinal cord injuries (Ji et al., 2017).

In our study, WBV significantly affected concentric extension and flexion muscle strength in athletes. The RCTs assessing these two variables employed the side-alternating mode of WBV, in which one foot was elevated relatively to the other, causing alternating movements between feet (Bidonde et al., 2017) and thus inducing rotational movements around the hip and lumbosacral joints (Rittweger et al., 2002). Such movements introduced an additional degree of freedom in the side-alternating WBV mode. Thus, whole-body mechanical impedance was lower during horizontal vibration than during synchronous vertical WBV, where vibration was applied simultaneously to both feet (Abercromby et al., 2007). None of the RCTs included complex WBV types, such as stochastic resonance WBV, or combined synchronous vertical and side-alternating vibrations. Stochastic resonance WBV is commonly used for older individuals because of its safety profiles; for example, it does not lead to exhaustion, and it helps maintain low blood pressure and lactate levels during training (de Bruin et al., 2020; Rogan and Taeymans, 2023).

Regarding the amplitude of WBV, our findings corroborate those of studies suggesting that an amplitude of 4 mm is optimal for clinical use (Al Masud et al., 2022; Stania et al., 2017).

### Muscle Power: CMJ Performance and SJ Height

The CMJ, a vertical jump, is used to assess power during training, for performance monitoring, and in research as an indicator of power output (Dobbs et al., 2015). During the CMJ, athletes flex their knees and hip joints to reach a quarter squat position before rapidly extending these joints to achieve the maximum jump height (Suchomel et al., 2016). The CMJ can be performed with or without an arm swing; nevertheless, incorporating the arm swing can enhance performance by >10% after WBV (Cheng et al., 2008; Feltner et al., 1999). CMJ performance is associated with maximal speed, maximal strength, and power. Jump height was reported to be a robust indicator of peak power (Cheng et al., 2008). Another variable commonly used to assess lower-body power is the SJ (Young, 1995). During the CMJ, the athlete moves downward from a standing position; this is immediately followed by an upward movement (takeoff). By contrast, during the SJ, the athlete descends into a semi-squat position and holds this position for approximately 3 s before the takeoff (Van Hooren and Zolotarjova, 2017). CMJ height tends to be greater than SJ height. This discrepancy may be attributable to the countermovement phase, which enables muscles to achieve increased levels of activity (fraction of attached cross-bridges) and force before the beginning of the shortening cycle; this enhances muscle performance during the initial phase of the jump (Bobbert et al., 1996). In our study, both CMJ height and SJ height exhibited non-significant increases after WBV. Because the control group received only conventional training, WBV might have exerted minimal or negligible additional effects on muscle power.

### Muscle Strength: Isometric and Concentric Torque of the Knee Extensors and Flexors

WBV has been reported to improve muscle strength of the knee extensors and flexors in athletes (Colson et al., 2010; Karatrantou et al., 2013; Wang et al., 2014). A study reported that vibration increased muscle activity in various lower-limb muscles; however, overall, no prominent dose-response relationship was observed between WBV acceleration or frequency and muscle response (Tankisheva et al., 2013). A meta-analysis indicated that WBV considerably improved strength of the knee extensors and flexors, hip extensors, and ankle plantar flexors in older adults (Gonçalves de Oliveira et al., 2023). A review highlighted that at high frequencies and amplitudes, WBV may be beneficial for training the quadriceps muscles (e.g., rectus femoris, vastus medialis, and vastus lateralis) and lower-limb posterior muscles (e.g., biceps femoris); however, the optimal frequency and amplitude remain to be determined (Al Masud et al., 2022). We observed significant improvements in the concentric torque of the knee extensors and flexors after WBV. However, isometric torque of these muscle groups exhibited no significant improvements. The improvement in strength may be attributable to the WBV-mediated activation of the TVR. Muscle stretching due to vibrations activates muscle spindles, eliciting a response similar to the traditional stretch reflex (Bosco et al., 1999). This increases electromyography activity (Di Giminiani et al., 2015; Ritzmann et al., 2010) and force production (Couto et al., 2012; Silva et al., 2008).

### Cardiovascular Endurance: VO2max

Athletic performance and overall fitness are extremely dependent on cardiovascular endurance, which enables athletes to effectively perform across various sports. VO_2max_, the gold standard for measuring aerobic fitness, assesses an athlete’s maximal oxygen utilization during intense exercise (LeMond and Hom, 2015). It serves as a reliable indicator of the cardiorespiratory system’s capacity to deliver oxygen to muscles during physical exertion under specific conditions of fitness and oxygen availability (Bertuzzi et al., 2013). VO_2max_ is correlated with performance in endurance activities such as distance running (Joyner and Coyle, 2008).

Our study revealed no significant improvements in VO_2max_ after WBV in athletes. This finding suggests that vibration exercises do not induce the same cardiovascular adaptations noted with conventional aerobic exercises. Our findings can be related to the fact that WBV may insufficiently enhance maximal aerobic power in trained athletes, possibly because the intensity of WBV training is typically <50% of VO_2max_ (Bertuzzi et al., 2013; Hurley et al., 1984). Our results are consistent with conclusions from a Cochrane review, which highlighted the lack of sufficient evidence supporting the potential of WBV to improve the heart rate and lung function compared with the benefits of conventional aerobic exercises (Bidonde et al., 2017).

### Strengths and Limitations

The strengths of the present study are its literature search strategy, which enabled retrieval of relevant RCTs conducted over a long period, and its assessment of performance indicators that may be influenced by WBV.

This study, however, has also some limitations. First, the quality of the included RCTs raised concerns; >50% (10/18) of the included studies presented some concerns or a high risk of bias. Furthermore, assessments based on the GRADE criteria revealed that the certainty of evidence was low or very low for all seven variables. The aforementioned factors likely reduced the reliability of our results. Second, some heterogeneity was observed among the RCTs because of variations in WBV settings (e.g., vibration amplitude, frequency, type, duration, and repetitions) and protocols (e.g., intervention duration, assessment time points, study country, and sport type). For example, a previous study has shown that a rest interval of 4 min resulted in significantly higher CMJ values than a rest interval of 2 min in highly trained karate practitioners (Pojskic et al., 2024). Moreover, differences in body composition and activity levels between men and women might have influenced the effects of WBV on exercise performance. Finally, the diverse range of sports included in the RCTs likely involved different exercise intensities; this discrepancy might have influenced our results.

## Conclusions

In our review of 18 RCTs, we investigated the effects of WBV on athletes’ exercise performance. Our meta-analysis revealed significant differences in the pooled estimates for the concentric torque of the knee extensors and flexors between the intervention and control groups. However, the overall quality of the evidence supporting these findings was low. Furthermore, we observed no significant post-intervention improvements in the indicators of power and cardiovascular endurance. Overall, our findings suggest that the current evidence is insufficient to enable provision of clear and generalized recommendations regarding the efficacy of WBV in improving athletes’ exercise performance. To provide evidence-based guidance for WBV, future studies should consider participants’ characteristics as well as intervention frequency, intensity, and duration in their analysis.

## Appendices

### Appendix 1A. PRISMA 2020 Checklist.

**Table:**

Section and Topic	Item #	Checklist item	Reported on page #
**Title**			
Title	1	Identify the report as a systematic review.	1
**Abstract**			
Abstract	2	See the PRISMA 2020 for Abstracts checklist.	2
**Introduction**			
Rationale	3	Describe the rationale for the review in the context of existing knowledge.	3
Objectives	4	Provide an explicit statement of the objective(s) or question(s) the review addresses.	3,4
**Methods**			
Eligibility criteria	5	Specify the inclusion and exclusion criteria for the review and how studies were grouped for the syntheses.	5
Information sources	6	Specify all databases, registers, websites, organisations, reference lists and other sources searched or consulted to identify studies. Specify the date when each source was last searched or consulted.	5
Search strategy	7	Present the full search strategies for all databases, registers and websites, including any filters and limits used.	5
Selection process	8	Specify the methods used to decide whether a study met the inclusion criteria of the review, including how many reviewers screened each record and each report retrieved, whether they worked independently, and if applicable, details of automation tools used in the process.	5
Data collection process	9	Specify the methods used to collect data from reports, including how many reviewers collected data from each report, whether they worked independently, any processes for obtaining or confirming data from study investigators, and if applicable, details of automation tools used in the process.	5
Data items	10a	List and define all outcomes for which data were sought. Specify whether all results that were compatible with each outcome domain in each study were sought (e.g. for all measures, time points, analyses), and if not, the methods used to decide which results to collect.	5
	10b	List and define all other variables for which data were sought (e.g. participant and intervention characteristics, funding sources). Describe any assumptions made about any missing or unclear information.	6
Study risk of bias assessment	11	Specify the methods used to assess risk of bias in the included studies, including details of the tool(s) used, how many reviewers assessed each study and whether they worked independently, and if applicable, details of automation tools used in the process.	6
Effect measures	12	Specify for each outcome the effect measure(s) (e.g. risk ratio, mean difference) used in the synthesis or presentation of results.	6
Synthesis methods	13a	Describe the processes used to decide which studies were eligible for each synthesis (e.g. tabulating the study intervention characteristics and comparing against the planned groups for each synthesis (item #5)).	6
	13b	Describe any methods required to prepare the data for presentation or synthesis, such as handling of missing summary statistics, or data conversions.	6
	13c	Describe any methods used to tabulate or visually display results of individual studies and syntheses.	6
	13d	Describe any methods used to synthesize results and provide a rationale for the choice(s). If meta-analysis was performed, describe the model(s), method(s) to identify the presence and extent of statistical heterogeneity, and software package(s) used.	6
	13e	Describe any methods used to explore possible causes of heterogeneity among study results (e.g. subgroup analysis, meta-regression).	6
	13f	Describe any sensitivity analyses conducted to assess robustness of the synthesized results.	6
Reporting bias assessment	14	Describe any methods used to assess risk of bias due to missing results in a synthesis (arising from reporting biases).	6
Certainty assessment	15	Describe any methods used to assess certainty (or confidence) in the body of evidence for an outcome.	6

### Appendix 1B. PRISMA 2020 Checklist.

**Table:**

Results			
Study selection	16a	Describe the results of the search and selection process, from the number of records identified in the search to the number of studies included in the review, ideally using a flow diagram.	6; figure 1
	16b	Cite studies that might appear to meet the inclusion criteria, but which were excluded, and explain why they were excluded.	6
Study characteristics	17	Cite each included study and present its characteristics.	6, 7; table 1, table 2
Risk of bias in studies	18	Present assessments of risk of bias for each included study.	7; figure 2
Results of individual studies	19	For all outcomes, present, for each study: (a) summary statistics for each group (where appropriate) and (b) an effect estimate and its precision (e.g. confidence/credible interval), ideally using structured tables or plots.	7,8; figure 3
Results of syntheses	20a	For each synthesis, briefly summarise the characteristics and risk of bias among contributing studies.	7,8; table 1, table2, figure 2
	20b	Present results of all statistical syntheses conducted. If meta-analysis was done, present for each the summary estimate and its precision (e.g. confidence/credible interval) and measures of statistical heterogeneity. If comparing groups, describe the direction of the effect.	7,8
	20c	Present results of all investigations of possible causes of heterogeneity among study results.	11
	20d	Present results of all sensitivity analyses conducted to assess the robustness of the synthesized results.	7,8
Reporting biases	21	Present assessments of risk of bias due to missing results (arising from reporting biases) for each synthesis assessed.	8
Certainty of evidence	22	Present assessments of certainty (or confidence) in the body of evidence for each outcome assessed.	8
**Discussion**			
Discussion	23a	Provide a general interpretation of the results in the context of other evidence.	9, 10
	23b	Discuss any limitations of the evidence included in the review.	11
	23c	Discuss any limitations of the review processes used.	11
	23d	Discuss implications of the results for practice, policy, and future research.	9–11
**Other information**			
Registration and protocol	24a	Provide registration information for the review, including register name and registration number, or state that the review was not registered.	5
	24b	Indicate where the review protocol can be accessed, or state that a protocol was not prepared.	5
	24c	Describe and explain any amendments to information provided at registration or in the protocol.	5
Support	25	Describe sources of financial or non-financial support for the review, and the role of the funders or sponsors in the review.	2
Competing interests	26	Declare any competing interests of review authors.	2
Availability of data, code and other materials	27	Report which of the following are publicly available and where they can be found: template data collection forms; data extracted from included studies; data used for all analyses; analytic code; any other materials used in the review.	2

### Appendix 2. Strategies used for database search.

**Table:**

Database	Search strategy and keywords
PubMed	(“Whole” [all fields] OR “wholeness” [all fields] OR “wholes” [all fields]) AND (“human body” [MeSH term] OR (“human” [all fields] AND “body” [all fields]) OR “human body” [all fields] OR “body” [all fields]) AND (“vibrate” [all fields] OR “vibrated” [all fields] OR “vibrates” [all fields] OR “vibrating” [all fields] OR “vibration” [MeSH term] OR “vibration” [all fields] OR “vibrations” [all fields] OR “vibrational” [all fields] OR “vibrator” [all fields] OR “vibrators” [all fields]) AND (“athlete s” [all fields] OR “athletes” [MeSH term] OR “athletes” [all fields] OR “athlete” [all fields] OR “athletically” [all fields] OR “athlets” [all fields] OR “sports” [MeSH term] OR “sports” [all fields] OR “athletic” [all fields] OR “athletics” [all fields] OR (“sport s” [all fields] OR “sports” [MeSH term] OR “sports” [all fields] OR “sport” [all fields] OR “sporting” [all fields]) OR (“exercise” [MeSH term] OR “exercise” [all fields] OR “exercises” [all fields] OR “exercise therapy” [MeSH term] OR (“exercise”[all fields] AND “therapy” [all fields]) OR “exercise therapy” [all fields] OR “exercising” [all fields] OR “exercise s” [all fields] OR “exercised” [all fields] OR “exerciser” [all fields] OR “exercisers” [all fields])). Restrictions: Title/abstract, clinical trial, full text, humans
Embase	(“Whole body vibration”/exp OR “whole body vibration” OR (“whole” AND (“body”/exp OR body) AND (“vibration”/exp OR vibration))) AND (“athletes”/exp OR athletes OR “sports”/exp OR sports OR “exercise”/exp OR exercise). Restrictions: Cochrane review, systematic review, meta-analysis, control clinical trial, randomized controlled trial
Cochrane	Whole body vibration AND (athletes OR sports OR exercise). Restrictions: Title/abstract/keyword, Cochrane review, trial, all dates
Chinese Electronic Periodical Services	Whole body vibration And (athletes OR sports OR exercise). Restrictions: None

*MeSH, Medical Subject Headings*

### Appendix 3. Protocols used for WBV in included studies.

**Table:**

Author(s)	Publication year	Intervention duration (weeks)	Intervention frequency (times/week)	Number of repetitions	Duration of each repetition (s)	Duration of rest between two repetition/set (s)
Annino et al.	2007	8	3	5	40	60
Arora et al.	2021	6	3	2 and 3 alternatively	NA	NA
Bertuzzi et al.	2013	6	2	3 and 6 alternatively	300	180
Celik et al.	2022	8	2	Weeks 3 and 6: 1 Other weeks: 2	30	30
Cheng et al.	2012	8	3	10	30 and 60 alternatively	60
Colson et al.	2010	4	3	20	30	30
Delecluse et al.	2005	5	2 to 3	3	30, 45, and 60 alternatively	60 s (for 30 s of each repetition), 20 s (for 45 s of each repetition), and 5 s (for 60 s of each repetition)
Fagnani et al.	2006	8	3	Weeks 1 and 2: 3	20 and 15 alternatively	60 s (for 20 s of each repetition) and 30 s (for 15 s of each repetition)
				Weeks 3 and 4: 3	30 and 20 alternatively	60 s (for 30 s of each repetition) and 30 s (for 20 s of each repetition)
				Weeks 5 and 6: 3	45 and 30 alternatively	45 s (for 45 s of each repetition) and 30 s (for 25 s of each repetition)
				Weeks 7 and 8: 4	60 and 30 alternatively	60 s (for 60 s of each repetition) and 30 s (for 30 s of each repetition)
Fort et al.	2012	15	3 to 4	7–10	30–60	60
Di Giminiani et al.	2009	8	3	8	20	240
Karatrantou et al.	2013	3	16 sessions in 2 weeks	2	300	120
Kvorning et al.	2006	9	Week 1: 1 Weeks 2 and 3: 2 Weeks 4–8: 3	6	30	120
Lamont et al.	2008	6	2	3	10	60
Martínez-Pardo et al.	2013	6	2	8–13	60	60
Oosthuyse et al.	2013	10	3	10	60	30
Roschel et al.	2015	6	2	3	30	180
Rubio-Arias et al.	2018	6	3	5	60	60
Wang et al.	2014	8	3	5	30	180

WBV, whole-body vibration; NA, not available

### Appendix 4. Body position and posture in included studies.

**Table:**

Author(s)	Publication year	Body position/posture
Annino et al.	2007	half-squat position (approximately 100) with feet and knee rotated externally
Arora et al.	2021	squats and heel raise
Bertuzzi et al.	2013	half-squat
Celik et al.	2022	lunge, squat, quadriceps stretch, deep squat, wide stance squat
Cheng et al.	2012	upright position with their knees flexed at the angle of 120
Colson et al.	2010	first, high squat position (knee angle of 110; where angle of 180 corresponds to full extension of the knee) and, second, the same high squat position while standing on the toes (with the same knee angle; where the ankle angle is fixed at the angle of 90)
Delecluse et al.	2005	high squat, deep squat, wide stance squat, one legged squat, lunge, calves
Fagnani et al.	2006	1. standing upright with the knee angle preset at an-angle-of-90 flexion, and the hands kept on the hips, 2. standing upright in the erect position with one leg on the vibration platform, the knee flexed at the angle of 90 and the other leg held in the air and hands on the hips
Fort et al.	2012	squat, leg press, leg curl, bench press, and pullover
Di Giminiani et al.	2009	participants stood on a platform at the angle of 90 between the lower and upper leg, while grasping a railing in front of them
Karatrantou et al.	2013	upright position with their knees flexed at the angle of 10
Kvorning et al.	2006	squat exercise was performed to the knee angle of 90
Lamont et al.	2008	Smith machine back squat
Martínez-Pardo et al.	2013	holding an isometric quarter squat position with the feet shoulder width apart
Oosthuyse et al.	2013	stood directly on the platform without any dampening
Roschel et al.	2015	half squats
Rubio-Arias et al.	2018	(1) one of their legs in front of the other, at a distance of 1 m between the support points of the feet (toes) and with the knees semi-flexed at the angle of 110–120; (2) squat position, on their toes, with their feet separated by about 50 cm and the knees flexed at the angle of 110–120; and (3) one-leg squat similar to the second exercise
Wang et al.	2014	knees at the angle of 120

WBV, whole-body vibration; NA, not available

### Appendix 5. GRADE criteria-based assessment of quality of evidence.

**Table:**

Certainty assessment							No. of patients		Effect		Certainty	Importance
No. of studies	Study design	Risk of bias	Inconsistency	Indirectness	Imprecision	Other considerations	WBV group	Control group	Relative (95% CI)	Absolute (95% CI)		
Countermovement jump performance												
10	RCT	Very serious^a^	Serious^b^	Not serious	Serious	None	135	110	-	MD: 0.66 (−0.13–1.44)	⨁◯◯◯ Very low	Important
Squat jump height												
6	RCT	Serious^d^	Serious^b^	Not Serious	Very Serious^e^	None	81	62	-	MD: 0.44 (−0.45–1.33)	⨁◯◯◯ Very low	Important
VO_2max_												
3	RCT	Serious^f^	Not Serious	Not Serious	Serious^c^	None	24	22	-	MD: −1.18 (−4.25–1.89)	⨁⨁◯◯ Low	Important
Isometric torque of the knee extensors												
4	RCT	Serious^g^	Serious^b^	Not Serious	Serious^c^	None	52	51	-	MD: 11.79 (−6.08–29.66)	⨁◯◯◯ Very low	Important
Isometric torque of the knee flexors												
3	RCT	Serious^h^	Not serious	Not serious	Serious^c^	None	35	36	-	MD: 6.19 (−1.32–13.69)	⨁⨁◯◯ Low	Important
Concentric torque of the knee extensors												
3	RCT	Serious^f^	Not serious	Not serious	Serious^i^	None	31	32	-	MD: 8.86 (6.00–11.72)	⨁⨁◯◯ Low	Important
Concentric torque of the knee flexors												
3	RCT	Serious^f^	Not serious	Not serious	Serious^i^	None	31	32	-	MD: 9.56 (7.40–11.72)	⨁⨁◯◯ Low	Important

CI, confidence interval; MD, mean difference; RCT, randomized controlled trial

#### Explanations

- 1 out of 10 RCTs had high risks and 4 out of 10 RCTs had some concerns
- Substantial heterogeneity (*I*^2^)
- Crossed the clinical decision threshold once
- 4 out of 6 RCTs had some concerns
- Crossed the clinical decision threshold twice
- 2 out of 3 RCTs had some concerns
- 1 out of 4 RCTs had some concerns
- 1 out of 3 RCTs had some concerns
- Sample size < 400
